# The feasibility for a novel minimally invasive surgery–percutaneous endoscopic transforaminal lumbar interbody fusion (PE-TLIF) for the treatment of lumbar degenerative diseases: a cadaveric experiment

**DOI:** 10.1186/s13018-020-01930-0

**Published:** 2020-09-08

**Authors:** Peng Yin, Yaoshen Zhang, Aixing Pan, Yi Ding, Liming Zhang, Chunyang Xu, Jincai Yang, Yong Hai

**Affiliations:** Department of Orthopaedics, Beijing Chao-Yang Hospital, China Capital Medical University, No. 8 GongTiNanLu, Chao-Yang District, Beijing, 100020 China

**Keywords:** Minimally invasive surgery, Percutaneous, Spinal endoscope, Transforaminal lumbar interbody fusion, Lumbar degenerative diseases

## Abstract

**Background:**

The objective of the study was to evaluate our innovative percutaneous endoscopic transforaminal lumbar interbody fusion (PE-TLIF) for the treatment of lumbar degenerative diseases.

**Methods:**

Two fresh-frozen human cadavers with soft tissues were donated for the experiment. Both cadavers had no history of previous spine surgery. The PE-TLIF surgery was performed on 3 levels (L4-5 of the first one, and L3-4, L4-5 of the second one) in October 2015. The PE-TLIF technique mainly included the following aspects: primary guide pins and a specially designed superior articular process (SAP) guide insertion, working channel setup, endoscopic decompression and fusion, and pedicle screw implantation and fixation. Under the surveillance of C-arm fluoroscope, four primary guide pins were inserted. The inferior primary guide in the hypothetically symptomatic side was confirmed as the first guide pin. At the end of the first guide pin, the specially designed SAP guide was installed. The secondary guide pin was inserted in the SAP via self-designed SAP guide. Under the protection cannula, part of the superior articular process was removed by oriented SAP resection device, so the working channel was smoothly put through the Kambin’s triangle. The endoscope was inserted close to the exiting nerve root. Rotation of the working channel kept the nerve root out of it.

**Results:**

Three levels of PE-TLIF were successfully performed in two cadavers. Self-designed SAP guide made the secondary guide pin inserting the SAP accurately. Decompression was adequate and the traversing nerve root was relieved. Three aimed intervertebral levels are implanted with two 7-mm-high PEEK cages and one expandable cage. The expandable cage could be adjusted from 8 mm to 13 mm. Surgical incisions included four 15 mm incisions for percutaneous screw fixation and one 12 mm incision for working channel. There was no nerve injury during the operations.

**Conclusions:**

Our present results showed that the novel minimally invasive surgery PE-TLIF was feasible for the treatment of lumbar degenerative diseases.

## Background

Spine fusion has been regarded as an effective treatment in improving pain, segment stability, function, and quality of life in patients with lumbar degenerative diseases [1–3]. Most of patients could acquire a satisfactory effect on the decompression of neural structures and stabilization for treated segments via conventional open lumbar fusion surgery; however, extensive destruction of posterior muscular-ligamentous complex usually leads to tremendous postoperative pain, muscular atrophy, and functional disability [4–7]. Hence, minimally invasive spine surgeries gradually gained popularity in the past 20 years. Although these minimally invasive surgeries could minimize injury to normal anatomic structures through tubular dilators for decompression and fusion [8–10], these techniques still require an open incision of the posterior muscular-ligamentous complex for tube placement.

Recently, endoscopic lumbar fusion techniques have been attempted in some studies [11–13], nevertheless, the total complication rate was 7.2–36%. Jacquot et al. believed that it was necessary to make decisive technical improvements to decrease the complication rate via endoscopic fusion surgeries [12]. With the advancement of endoscopic fusion techniques, some researchers have reported satisfactory outcomes through endoscopic surgery, but the learning curve of these techniques was relatively long [14, 15]. Hence, we developed a percutaneous endoscopic transforaminal lumbar interbody fusion (PE-TLIF) technique. The technique mainly included newly oriented superior articular process (SAP) resection device, parallel expandable cage, and improved working channel in order to shorten the learning curve and hopefully decrease the complication rate. This study aimed to investigate the feasibility of our endoscopic technique for the treatment of lumbar degenerative diseases on frozen cadavers.

## Method

Two fresh-frozen human cadavers with soft tissues were donated for the experiment (Beijing Chaoyang Hospital). Both cadavers had no history of previous spine surgery. The PE-TLIF surgery was performed on 3 levels (L4-5 of the first one, and L3-4, L4-5 of the second one) in October 2015. The experiment study was approved by the institutional review board of Beijing Chaoyang Hospital.

### Surgical technique

#### Primary guide pins and a specially designed SAP guide insertion

**Fig. 1:**
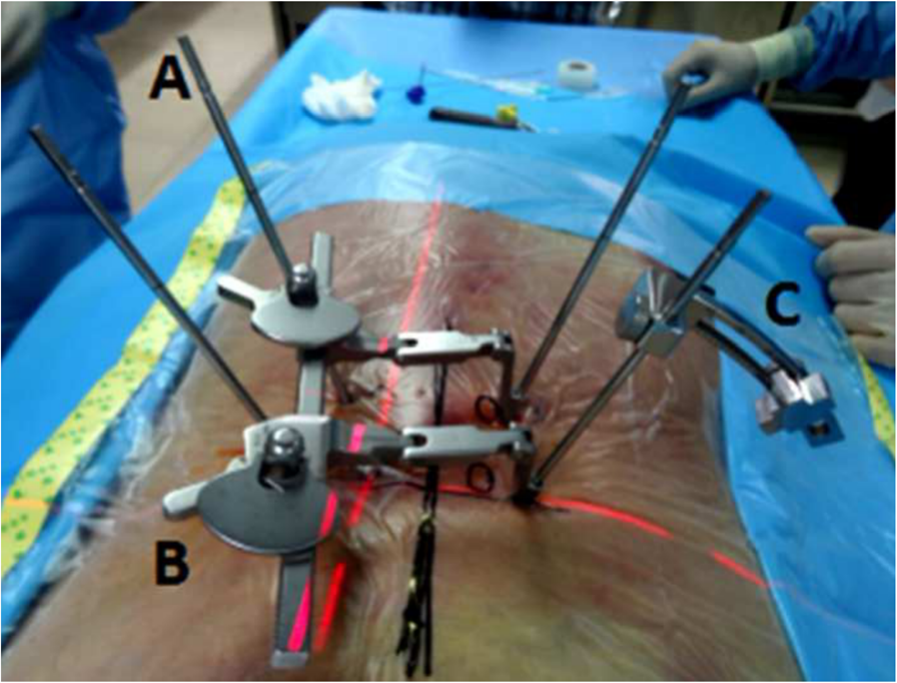
The schematic diagram of the primary guide pin (**a**), retractor (**b**), and specially designed SAP guide (**c**)

The cadavers were positioned prone on a radiolucent table. The aimed lumbar segment was confirmed under the C-arm fluoroscope. After disinfection, the body was draped in a sterile fashion. Syringe needles were used to identify the pedicle positions. Four incisions (5 mm) were made and then 4 primary guide pins were inserted. The depth was determined by fluoroscopy. The inferior primary guide in the hypothetically symptomatic side was confirmed as the first guide pin. At the end of the first guide pin, the specially designed SAP guide was installed (Fig. 1).

### Working channel setup

**Fig. 2:**
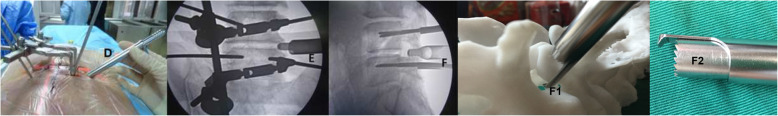
**D** Protection cannula. **E** Protection cannula under the anterior-posterior X-ray film. **F** Protection cannula under the lateral X-ray film. The hook-shaped device front is attached to the SAP (**F1**), then the SAP been taken out by the ring saw (**F2**)

With the guidance of the SAP guide, a secondary guide pin was placed at the superior articular process percutaneously. Then the guide was removed, and a 12-mm skin incision was made along the secondary guide pin. Dilating and protection cannulas were inserted progressively with the help of secondary guide pin (Fig. 2). While soft tissues and nerves were protected by the protection cannula, the part of the superior articular process was excised and taken out using a ring saw. With the guidance of a guide rod, working channel with 10 mm inner diameter was deployed through Kambin’s triangle.

### Endoscopic decompression and fusion

**Fig. 3:**
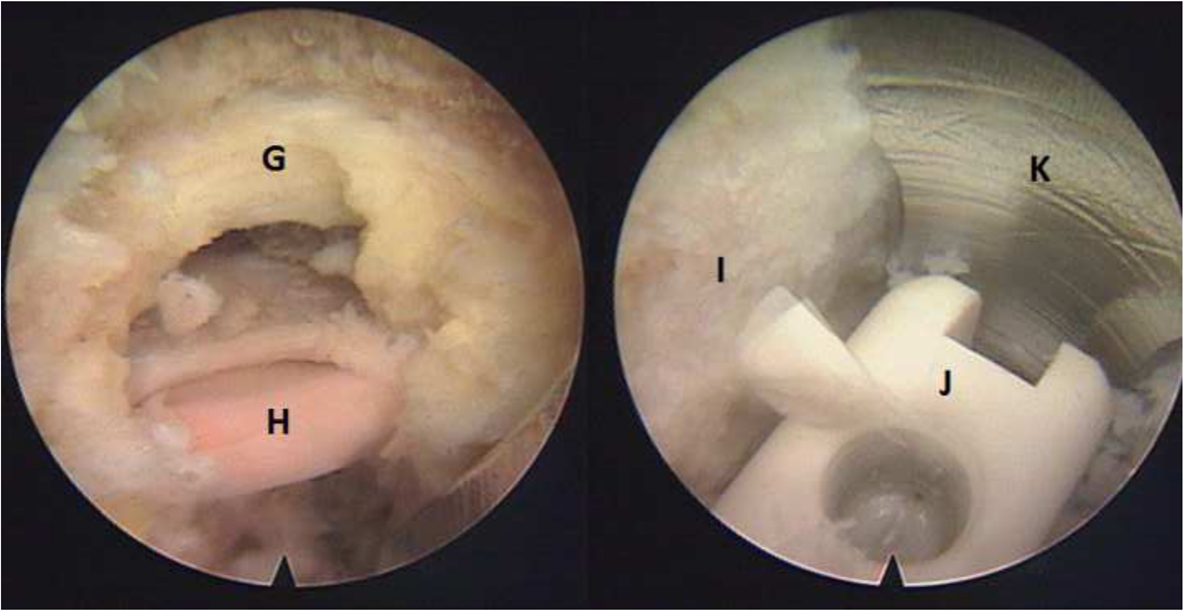
G, the part of the superior articular process were removed. H, traversing nerve root under the endoscope. I, superior endplate. J, fusion cage under the endoscope. K, the front tip of protection cannula

The endoscope was connected and the working channel was moved right to the intervertebral disc. Protection cannula was rotated to keep the exiting nerve root safe (Fig. 3). Under endoscopic monitoring, ligament flavum dissection was performed; the remaining superior articular process was removed by micro scissors or a burr drill. Then, the lateral spinal canal was decompressed and the traversing nerve root was released. After confirming that the traversing and exiting nerve roots were protected out of the working channel via endoscopic vision, the endoscope was taken out temporarily and the discectomy was performed. The cartilaginous endplates of the vertebral bodies were scraped away and the endoscope was installed again to check the intervertebral space. After the endplates were prepared adequately, the endoscope was removed and the fusion cage (7 mm height PEEK or Titanium expandable) was inserted through the working channel under radioscopy. The spinal canal was checked with the endoscope making sure that the nerve root was relieved.

### Pedicle screw implantation and fixation

**Fig. 4:**
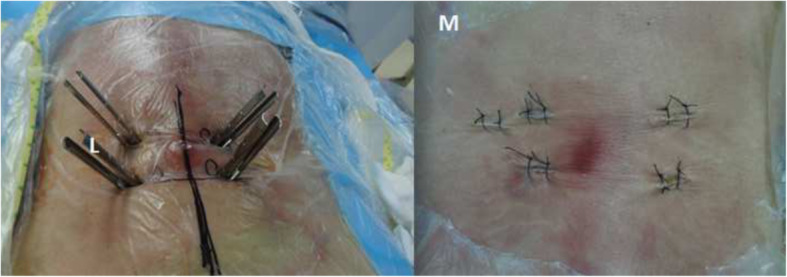
**L** Pedicle screws were inserted percutaneously. **M** The appearance of incisions

**Fig. 5:**
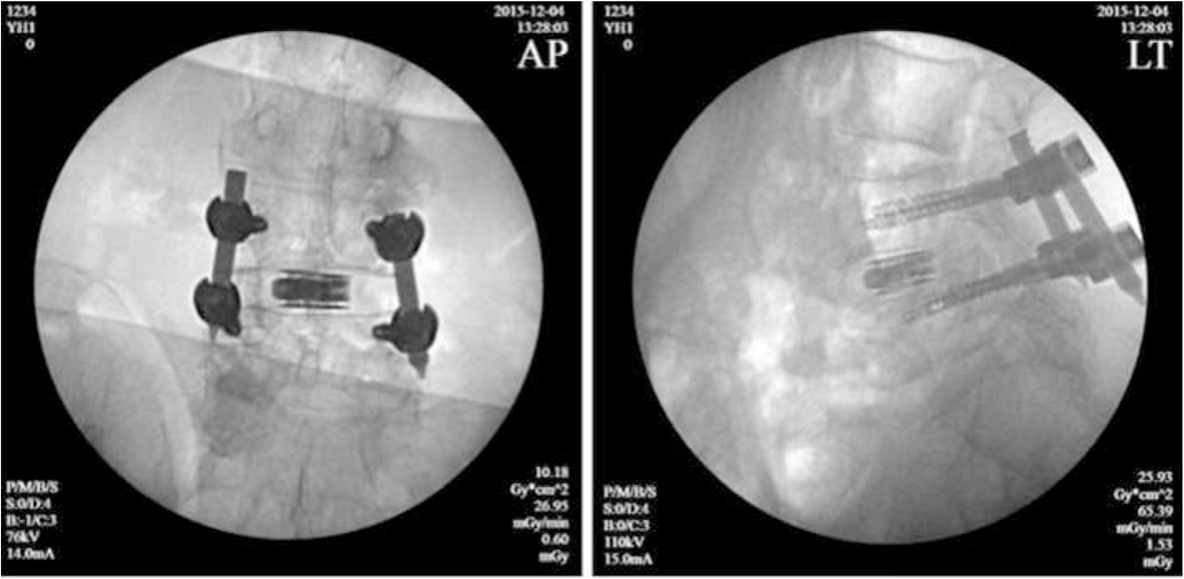
X-ray images showed the position of internal fixation and cage were favorable

Finally, the primary pins were replaced by guide wires and four pedicle screws were implanted into the planned positions with the help of the radioscopic device. Two rods were inserted percutaneously and the screw-rod attachment was tightened. At last, the skin was sutured and the position of screws and cage were re-checked by C-arm fluoroscope (Figs. 4 and 5).

### The primary outcomes

The primary outcomes included accuracy of the secondary guide pin insertion, safety of oriented SAP resection, and effectiveness of decompression. Secondary outcomes included surgical incision lengths and related complications.

## Results

Three levels of PE-TLIF were successfully performed. Self-designed SAP guide made the secondary guide pin inserting the SAP accurately. Under the protection cannula, part of the SAP was removed by oriented SAP resection device, so the working channel was smoothly put through the Kambin’s triangle. Decompression was adequate and the traversing nerve root was relieved. Since both traversing and exiting nerve roots were confirmed under the endoscope and both types of nerve were protected outside of the working channel, and then we could safely perform the discectomy and fusion cage insertion without endoscopic monitoring. Three aimed intervertebral levels were implanted with two 7-mm-high PEEK cages and one expandable cage. The expandable cage could be adjusted from 8 mm to 13 mm. Surgical incisions included four 15 mm incisions for percutaneous screw fixation and one 12 mm incision for working channel. There was no nerve injury during the surgeries.

## Discussion

Our study showed that the PE-TLIF technique could be feasible for the treatment of lumbar degenerative diseases, and related surgical instruments were able to complete the surgery on the human cadavers successfully.

In 2012, Said et al. firstly reported endoscopic fusion techniques for the treatment of lumbar degenerative diseases. Although most of treatment outcomes were satisfactory, the total complication rate was up to 20% [11]. And then, Jacquot et al. demonstrated that the complication rate was up to 36% via endoscopic lumbar interbody fusion, and they believed that technical improvements were necessary [12]. After that, in order to decrease the complication rate, several researchers began to design and improve the endoscopic lumbar interbody fusion and related surgical instruments [11–19]. Most of studies showed that the endoscopic lumbar fusion technique could be a promising treatment for lumbar degenerative diseases. The fusion rate was 59.6–100%, and the complication rate was 0–36%. However, there were no standard operating procedures and uniform indications on lumbar degenerative diseases. More details were listed in Tables 1 and 2.

**Table 1:** Characteristics of included studies

Author	Study no.	Year	Journal	Study design	Number of patients	Age (years)	Male/ female	Indication	Follow-up (months)
Osman et al.	1	2012	International Journal of Spine Surgery	RS	60	52.8 (26–85)	30/30	DDD (8.3%) LSS (81.7%) SL (10%)	12 (6-25)
Jacquot et al.	2	2013	International Orthopaedics	RS	57	50.29 (34–71) Male 57.42 (29–90) Female	17/40	DDD (100%) PO (33%)	24
He et al.	3	2015	International Journal of Surgery	RS	42	64.2 ± 12.8 (37–75)	23/19	LSS (81.0%) DSL (14.3%) LDH (4.8%)	27.6 ± 3.8 (24-36)
Morgenstern et al.	4	2015	International Journal of Spine Surgery	RS	30	62.2 ± 15.9	12/18	DDD (30%) SL (40%) FA (20%) IAD (6.67%) CD (3.33%)	38 ± 17 (11-67)
Wang et al.	5	2016	Neurosurgical Focus	RS	10	62.2 ± 9.0 (52–78)	7/3	DDD (100%) SL (60%)	12
Lee et al.	6	2017	BioMed Research International	RS	18	44.1 (26–63)	None	DDD (88.9%) SL (11.1%)	46 (12-123)
Heo et al.	7	2017	Neurosurgical Focus	RS	69	71.2 ± 7.8	24/45	SL (87.0%) LSS (13%)	13.5±7.1
Kim et al.	8	2018	Clinics in Orthopedic Surgery	RS	14	68.7 ± 8.5 (49–85)	None	LSS (57.1%) SL (42.9%)	2
Wu et al.	9	2018	BioMed Research International	RS	7	56.0 ± 13.0 (33–72)	3/3	SL (100%)	35.1±3.0 (31.5-38.1)

*RS* retrospective case series, *DDD* degenerative disc disease, *LSS* lumbar spinal stenosis, *SL* spondylolisthesis, *PO* previous operation, *DSL* degenerative spondylolisthesis, *FA* failed arthrodesis, *IAD* instability after decompression, *CD* chondroma

**Table 2:** Interventions and outcomes of included studies

Study no.	Technique	Fusion level	Operation time (min)	Blood loss (ml)	The length of hospital stay (days)	Clinical effects	Complications	Fusion rate
1	ETD LIF PPSI	L1/2 L2/3 L3/4 L4/5 L5/S1	174 (117–251)	57.6 (30–100)	2.6 (1–12)	All patients improve on VAS and RMDQ	8 patients RSE 2 patients RN 2 patients PSC 20%	59.6%
2	PETLIF	L3/4 L4/5 L5/S1	60 ± 30	None	5 (2–21)	43.9% patients improve on VAS and ODI	8 patients RPP 13 patients AMC 36%	77%
3	FE-MISTLIF	L3/4 L4/5 L5/S1	133.9 ± 16.1 one segment 241.3 ± 36.5 Two segments	221.8 ± 98.5 (100–550)	9.6 ± 1.3 (7–12)	All patients improve on VAS and ODI success rate 95.2%	2 patients PNC	92.9%
4	PTLIF	L2/3 L3/4 L4/5 L5/S1	120 ± 30 (group A or B) 240 ± 120 (group C)	None	1.1 (0.8–2.8)	All patients improve on VAS and ODI	3 patients TD 2 patients SIP	None
5	E-MISTLIF	None	113.5 ± 6.3 (105–120)	65 ± 38 (30–190)	1.4 ± 1.3	90% patients improve on ODI, SF-36, EQ-5D	No complications	None
6	PTLIF	L2/3 L4/5 L5/S1	77 (62–100)	None	1.0 (0.5–2.1)	All patients improve on VAS and ODI	1 patient PNC 1 patient nonunion 1 patient revision	88.9%
7	UBE	L3/4 L4/5 L5/S1	165.8 ± 25.5	85.5 ± 19.41	None	All patients improve on VAS and ODI	2 patients DT 3 patients PEH	None
8	BE-TLIF	L3/4 L4/5 L5/S1	169 ± 10	74 ± 9	None	All patients improve on VAS	1 patient L5 Paralysis 1 patient DT	None
9	PELIF	L4/5	167.5 ± 30.9 (135–220)	70.0 ± 24.5 (50–100)	1.2 ± 0.6	All patients improve on VAS, SF-36 and ODI	No complications	100%

*VAS* visual analog scale, *RMDQ* Roland-Morris Disability Questionnaire, *ETD* endoscopic transforaminal decompression, *LIF* lumbar interbody fusion, *PPSI* percutaneous pedicle screw implantation, *RSE* residual discomfort on extension, *RN* residual numbness PSC pedicle screw-related complications, *PETLIF* percutaneous endoscopic transforaminal lumbar interbody fusion, *RPP* radicular pain with paresthesias, *AMC* asymptomatic migration of the cages, *ODI* Oswestry disability index, *FE-MISTLIF* full-endoscopic minimally invasive transforaminal lumbar interbody fusion, *PNC* postoperative neurological complications, *PTLIF* percutaneous transforaminal lumbar interbody fusion, *TD* transitory dysesthesia, *SIP* sacroiliac pain, *SF-36* 36-Item Short Form Health Survey, *UBE* unilateral biportal endoscopic technique, *DT* dural tear, *PEH* postoperative epidural hematoma, *BE* biportal endoscopic

The major advantage of our technique was to safely and effectively resect the SAP. Design of the oriented SAP resection device is based on the relative constant anatomy relation between SAP and pedicles in lumbar spine, so the part of SAP could be removed without nerve injury as long as the standard procedure was performed. Meanwhile, the depth of incision could be restricted by the hook-shaped device in front of the cannula for SAP resection which would prevent exiting nerve root and dura mater from trepan-cutting. Furthermore, a meticulous preoperative evaluation of individual relations among SAP and the surrounding structures on MRI and CT scan are also needed to ensure a safe and efficient resection. We also improved the diameter of the working channel to protect exiting and traversing nerve roots, and for the benefit of cage insertion via percutaneous surgery. Under the auxiliary of protection tube, facet arthroplasty in Kambin triangle was achieved with the help of guided SAP resection device. In the view of the endoscope, we could observe the existing and traversing nerve roots clearly. Spinal canal decompression could be done without insult to the nerve roots as we could find the anatomic structure clearly. After nerve decompression and complete endplate preparation, a 7-mm-high PEEK cage or an adjustable diameter parallel expandable cage (7 mm–13 mm) was inserted into the interbody space. We would like to recommend the parallel expandable cage for lumbar endoscopic fusion. We believed that expandable cages provided an instant stability of lumbar spine with the assist of an adjustable diameter, and intervertebral space height was restored high enough to offer an indirect decompression of confined lateral recess. Only 5 small incisions ranged from 10 mm to 15 mm were needed. Our technique caused minor damage to paravertebral muscles, ligaments, and bony structures. In the meantime, our technique could theoretically decrease intraoperative blood loss and preserves posterior spine structure, making a faster postoperative recovery.

## Conclusion

The percutaneous endoscopic transforaminal lumbar interbody fusion (PE-TLIF) is feasible, and the related surgical instruments could be able to complete the trial surgery on the human cadavers successfully. However, at present, this study is still only on the stage of simulated operation on human cadavers. It is necessary to perform further clinical experiment. Besides, the related surgical instruments also need to be improved continuously. Our present results showed that the novel minimally invasive surgery PE-TLIF was feasible for the treatment of lumbar degenerative diseases.

## Data Availability

All data were included in the manuscript.

## Abbreviations

PE-TLIF: Percutaneous endoscopic transforaminal lumbar interbody fusion
SAP: Superior articular process
